# Supplementary material for: Publication bias in otorhinolaryngology meta-analyses in 2021
Source: Syst Rev. 2024 Jan 2;13:11. doi: 10.1186/s13643-023-02404-0 (PMC10762811; doi:10.1186/s13643-023-02404-0)
Supplement: Supplementary file 1 — Additional file 1: Appendix. The title, journal, and type of the review of the 75 systematic reviews included in this study are as follows. [file 13643_2023_2404_MOESM1_ESM.pdf]

Appendix

The title, journal, and type of the review of the 75 systematic reviews included in this study [1–75] are as follows:

Journal	Title	Review type
Audiol. Neurootol.	Crucial Music Components Needed for Speech Perception Enhancement of Pediatric Cochlear Implant Users: A Systematic Review and Meta-Analysis	Effectiveness
Ear Hear	Occupational Hearing Loss Associated With Non-Gaussian Noise: A Systematic Review and Meta-analysis	Etiology and/or Risk
Ear Hear	Prevalence of Decreased Sound Tolerance (Hyperacusis) in Individuals With Autism Spectrum Disorder	Prevalence and/or Incidence
Int Forum Allergy Rhinol.	Effects of olfactory training on posttraumatic olfactory dysfunction: a systematic review and meta-analysis	Effectiveness
Int Forum Allergy Rhinol.	Effects of implants with steroids after endoscopic sinus surgery: A systematic review with meta-analysis of randomized controlled trials	Effectiveness
Int Forum Allergy Rhinol.	Usefulness of intraoperative frozen section for diagnosing acute invasive fungal rhinosinusitis: A systematic review and meta-analysis	Effectiveness
Int Forum Allergy Rhinol.	Topical nasal treatment efficacy on adult obstructive sleep apnea severity: a systematic review and meta-analysis	Diagnostic Test Accuracy
Int Forum Allergy Rhinol.	Botulinum toxin for chronic rhinitis: A systematic review and meta-analysis	Etiology and/or Risk
Int Forum Allergy Rhinol.	HPV in the malignant transformation of sinonasal inverted papillomas: A meta-analysis	Effectiveness
Otolaryngol Head Neck Surg.	Complications After Soft Tissue With Plate vs Bony Mandibular Reconstruction: A Systematic Review and Meta-analysis	Etiology and/or Risk
Otolaryngol Head Neck Surg.	Prognostic Significance of Extranodal Extension in HPV-Mediated Oropharyngeal Carcinoma: A Systematic Review and Meta-analysis	Etiology and/or Risk
Otolaryngol Head Neck Surg.	Tracheotomy in COVID-19 Patients: A Systematic Review and Meta-analysis of Weaning, Decannulation, and Survival	Effectiveness
Otolaryngol Head Neck Surg.	Systematic Review of Second Primary Oropharyngeal Cancers in Patients With p16+ Oropharyngeal Cancer	Prevalence and/or Incidence
Otolaryngol Head Neck Surg.	Systematic Review and Meta-analysis of the Change in Pharyngeal Bacterial Cultures After Pediatric Tonsillectomy	Effectiveness
Otolaryngol Head Neck Surg.	Pectoralis Major Onlay vs Interpositional Reconstruction Fistulation After Salvage Total Laryngectomy: Systematic Review and Meta-analysis	Effectiveness
Otolaryngol Head Neck Surg.	Meta-analysis Exploring Sinopulmonary Outcomes of Aspirin Desensitization in Aspirin-Exacerbated Respiratory Disease	Effectiveness
Otolaryngol Head Neck Surg.	Velcro Ties in Early Postoperative Pediatric Tracheostomy Care: A Systematic Review and Meta-analysis	Etiology and/or Risk
Otolaryngol Head Neck Surg.	Idiopathic Sudden Sensorineural Hearing Loss in Children: A Systematic Review and Meta-analysis	Etiology and/or Risk
Otolaryngol Head Neck Surg.	Systematic Review and Meta-analysis of Endoscopic vs Microscopic Stapes Surgery for Stapes Fixation	Effectiveness
Otolaryngol Head Neck Surg.	High-Risk Human Papillomavirus–Related Oropharyngeal Squamous Cell Carcinoma Among Non-Indigenous and Indigenous Populations: A Systematic Review	Etiology and/or Risk
Otolaryngol Head Neck Surg.	Olfactory Training for Postviral Olfactory Dysfunction: Systematic Review and Meta-analysis	Prevalence and/or Incidence
Otolaryngol Head Neck Surg.	Systematic Review and Meta-analysis: Effectiveness of Corticosteroids in Treating Adults With Acute Vestibular Neuritis	Effectiveness
Otolaryngol Head Neck Surg.	Antibiotic Prophylaxis for Thyroid and Parathyroid Surgery: A Systematic Review and Meta-analysis	Effectiveness
Otolaryngol Head Neck Surg.	Sinogenic Intracranial Suppuration in Children: Systematic Review and Meta-analysis	Etiology and/or Risk
Otolaryngol Head Neck Surg.	Factors Influencing the Development of Pneumonia in Patients With Head and Neck Cancer: A Meta-analysis	Effectiveness
Otolaryngol Head Neck Surg.	Prevalence and Characteristics of Taste Disorders in Cases of COVID-19: A Meta-analysis of 29,349 Patients	Effectiveness
Otolaryngol Head Neck Surg.	Surgical Management of Sialorrhea: A Systematic Review and Meta-analysis	Prognostic

Otolaryngol Head Neck Surg.	Surgical Management of Bilateral Vocal Fold Paralysis in Children: A Systematic Review and Meta-analysis	Prognostic
Otolaryngol Head Neck Surg.	Management of Type 1 Laryngeal Clefts: A Systematic Review and Meta-analysis	Prevalence and/or Incidence
Otolaryngol Head Neck Surg.	Influence of Surgical Techniques on Endoscopic Dacryocystorhinostomy: A Systematic Review and Meta-analysis	Prevalence and/or Incidence
Otolaryngol Head Neck Surg.	Management of Flap Failure After Head and Neck Reconstruction: A Systematic Review and Meta-analysis	Effectiveness
Rhinology	Elective neck irradiation in the management of esthesioneuroblastoma: a systematic review and meta-analysis	Etiology and/or Risk
Rhinology	Intralymphatic immunotherapy for allergic rhinoconjunctivitis: a systematic review and meta-analysis	Effectiveness
Rhinology	Clinical effectiveness of house dust mite immunotherapy in mono- versus poly-sensitised patients with allergic rhinitis: a systematic review and meta-analysis	Prognostic
Rhinology	Anxiety and depression risk in patients with allergic rhinitis: a systematic review and meta-analysis	Effectiveness
Rhinology	Leukotriene receptor antagonist addition to intranasal steroid: systematic review and meta-analysis	Effectiveness
Rhinology	Is postoperative nasal packing after septoplasty safe? A systematic review and meta-analysis of randomized controlled studies	Etiology and/or Risk
Rhinology	Omalizumab for the treatment of allergic rhinitis: a systematic review and meta-analysis	Effectiveness
Laryngoscope	Tracheal Resection in the Management of Thyroid Cancer: An Evidence-Based Approach	Effectiveness
Laryngoscope	Autofluorescence and Indocyanine Green in Thyroid Surgery: A Systematic Review and Meta-Analysis	Prevalence and/or Incidence
Laryngoscope	Treatment of Vestibular Migraine: A Systematic Review and Meta-analysis	Effectiveness
Laryngoscope	Endoscopy-Assisted Transoral Approach to Resect Parapharyngeal Space Tumors: A Systematic Review and Meta-Analysis	Etiology and/or Risk
Laryngoscope	Outcomes of Adenotonsillectomy for Obstructive Sleep Apnea in Prader-Willi Syndrome: Systematic Review and Meta-analysis	Effectiveness
Laryngoscope	Sialendoscopy and Sjogren's Disease: A Systematic Review	Effectiveness
Laryngoscope	The Relationship between Croup and Gastroesophageal Reflux: A Systematic Review and Meta-Analysis	Etiology and/or Risk
Laryngoscope	Different Surgical Strategies in the Prevention of Frey Syndrome: A Systematic Review and Meta-analysis	Etiology and/or Risk
Laryngoscope	Association Between Human Papilloma Virus Infection and Malignant Sinonasal Inverted Papilloma	Prevalence and/or Incidence
Laryngoscope	Neuromodulators for Atypical Facial Pain and Neuralgias: A Systematic Review and Meta-Analysis	Effectiveness
Laryngoscope	Risk Factors for Multiple Tympanostomy Tube Placements in Children: Systematic Review and Meta-Analysis	Effectiveness
Laryngoscope	Lipoinjection for Unilateral Vocal Fold Paralysis Treatment: A Systematic Review and Meta-Analysis	Etiology and/or Risk
Laryngoscope	Pharmacological Treatments of Bell's Palsy in Adults: A Systematic Review and Network Meta-Analysis	Etiology and/or Risk
Laryngoscope	Scalar Translocation Comparison Between Lateral Wall and Perimodiolar Cochlear Implant Arrays - A Meta-Analysis	Etiology and/or Risk
Laryngoscope	Effect of Sleep Surgery on C-Reactive Protein Levels in Adults With Obstructive Sleep Apnea: A Meta-Analysis	Etiology and/or Risk
Laryngoscope	Method of Lateral Osteotomy to Reduce Eyelid Edema and Ecchymosis After Rhinoplasty: A Meta-analysis	Effectiveness
Laryngoscope	Usefulness of Sentinel Lymph Node Biopsy for Oral Cancer: A Systematic Review and Meta-Analysis	Etiology and/or Risk
Laryngoscope	Risk of Stroke and Myocardial Infarction After Sudden Sensorineural Hearing Loss: A Meta-Analysis	Etiology and/or Risk
Laryngoscope	Survival of Young Versus Old Patients With Oral Cavity Squamous Cell Carcinoma: A Meta-Analysis	Prognostic
Laryngoscope	A Meta-Analysis of 67 Studies with Presenting Symptoms and Laboratory Tests of COVID-19 Patients	Effectiveness
Laryngoscope	Nasal Peak Inspiratory Flow in Healthy and Obstructed Patients: Systematic Review and Meta-Analysis	Etiology and/or Risk

Laryngoscope	Three-Dimensional Endoscopic Endonasal Surgery: A Systematic Review	Prevalence and/or Incidence
Laryngoscope	Barbed Reposition Pharyngoplasty versus Expansion Sphincter Pharyngoplasty: A Meta-Analysis	Effectiveness
Laryngoscope	Misperception of Visual Vertical in Peripheral Vestibular Disorders. A Systematic Review With Meta-Analysis	Effectiveness
Laryngoscope	Prevalence of Olfactory Dysfunction in Coronavirus Disease 2019 (COVID-19): A Meta-analysis of 27,492 Patients	Effectiveness
Laryngoscope	Paper Patching Versus Watchful Waiting of Traumatic Tympanic Membrane Perforations: A Meta-Analysis	Effectiveness
Laryngoscope	Residual Perforation Risk Assessment of Intratympanic Steroids via Tympanostomy Tube Versus Transtympanic Injections	Etiology and/or Risk
Laryngoscope	Complications of Neck Drains in Thyroidectomies: A Systematic Review and Meta-Analysis	Effectiveness
Laryngoscope	Bleeding Complications After Transoral Robotic Surgery: A Meta-Analysis and Systematic Review	Effectiveness
Laryngoscope	Prevalence of Sensorineural Hearing Loss in Pediatric Patients with Sickle Cell Disease: A Meta-analysis	Etiology and/or Risk
Laryngoscope	A Systematic Review and Meta-Analysis: Timing of Elective Removal of Tympanostomy Tubes	Etiology and/or Risk
Laryngoscope	Cochlear Implantation in Meniere's Disease: A Systematic Review and Meta-Analysis	Diagnostic Test Accuracy
Laryngoscope	Radiologically Defined Sarcopenia Affects Survival in Head and Neck Cancer: A Meta-Analysis	Prevalence and/or Incidence
Laryngoscope	Sudden Sensorineural Hearing Loss in Children—Management and Outcomes: A Meta-analysis	Effectiveness
Laryngoscope	Slide Tracheoplasty for Congenital Tracheal Stenosis Repair: A Systematic Review and Meta-Analysis	Etiology and/or Risk
Laryngoscope	A Systematic Review and Meta-Analysis of Taste Dysfunction in Chronic Rhinosinusitis	Prognostic
Laryngoscope	Effect of Endoscope Sinus Surgery on Pulmonary Function in Cystic Fibrosis Patients: A Meta-Analysis	Prevalence and/or Incidence

References

1. Chang, B. A., Gurberg, J., Ware, E. & Luu, K. Velcro Ties in Early Postoperative Pediatric Tracheostomy Care: A Systematic Review and Meta-analysis. *Otolaryngology–Head and Neck Surgery* **164**, 1148–1152 (2021).
2. Kattar, N. *et al.* Olfactory Training for Postviral Olfactory Dysfunction: Systematic Review and Meta-analysis. *Otolaryngology–Head and Neck Surgery* **164**, 244–254 (2021).
3. Neruntarat, C., Khuancharee, K. & Saengthong, P. Barbed Reposition Pharyngoplasty versus Expansion Sphincter Pharyngoplasty: A Meta-Analysis. *Laryngoscope* **131**, 1420–1428 (2021).
4. Stokes, W. *et al.* Bleeding Complications After Transoral Robotic Surgery: A Meta-Analysis and Systematic Review. *Laryngoscope* **131**, 95–105 (2021).
5. Brietzke, S. E. & Andreoli, S. M. Systematic Review and Meta-analysis of the Change in Pharyngeal Bacterial Cultures After Pediatric Tonsillectomy. *Otolaryngology–Head and Neck Surgery* **164**, 264–270 (2021).
6. Jwair, S. *et al.* Scalar Translocation Comparison Between Lateral Wall and Perimodiolar Cochlear Implant Arrays - A Meta-Analysis. *Laryngoscope* **131**, 1358–1368 (2021).
7. Leong, K.-J., Lau, T., Stewart, V. & Canetti, E. F. D. Systematic Review and Meta-analysis: Effectiveness of Corticosteroids in Treating Adults With Acute Vestibular Neuritis. *Otolaryngology–Head and Neck Surgery* **165**, 255–266 (2021).
8. Nguyen, D., Liang, J. & Durr, M. Topical nasal treatment efficacy on adult obstructive sleep apnea severity: a systematic review and meta-analysis. *International Forum of Allergy & Rhinology* **11**, 153–161 (2021).

9. Seresirikachorn, K., Mullol, J., Limitlaohaphan, K., Asvapoositkul, V. & Snidvongs, K. Leukotriene receptor antagonist addition to intranasal steroid: systematic review and meta-analysis. *Rhinology journal* **59**, 2–9 (2021).
10. Xie, D. X., Leland, E. M., Seal, S. M., Lin, S. Y. & Rowan, N. R. A Systematic Review and Meta-Analysis of Taste Dysfunction in Chronic Rhinosinusitis. *Laryngoscope* **131**, 482–489 (2021).
11. Coca, K. K. *et al.* Sialendoscopy and Sjogren's Disease: A Systematic Review. *Laryngoscope* **131**, 1474–1481 (2021).
12. Huang, T., Wei, Y. & Wu, D. Effects of olfactory training on posttraumatic olfactory dysfunction: a systematic review and meta-analysis. *International Forum of Allergy & Rhinology* **11**, 1102–1112 (2021).
13. Kim, J., Kim, S. H. & Hwang, S. H. Method of Lateral Osteotomy to Reduce Eyelid Edema and Ecchymosis After Rhinoplasty: A Meta-analysis. *Laryngoscope* **131**, 54–58 (2021).
14. Tan, G. X., Hamilton, A. & MacArthur, C. J. A Systematic Review and Meta-Analysis: Timing of Elective Removal of Tympanostomy Tubes. *Laryngoscope* (2021) doi:10.1002/lary.30003.
15. Lammers, M. J. W., Young, E., Westerberg, B. D. & Lea, J. Risk of Stroke and Myocardial Infarction After Sudden Sensorineural Hearing Loss: A Meta-Analysis. *Laryngoscope* **131**, 1369–1377 (2021).
16. Rinzin, K., Hoang, M. P., Seresirikachorn, K. & Snidvongs, K. Botulinum toxin for chronic rhinitis: A systematic review and meta-analysis. *International Forum of Allergy & Rhinology* **11**, 1538–1548 (2021).
17. Hoang, M. P., Seresirikachorn, K., Chitsuthipakorn, W. & Snidvongs, K. Intralymphatic immunotherapy for allergic rhinoconjunctivitis: a systematic review and meta-analysis. *Rhinology journal* **0**, 0–0 (2021).
18. Franz, L., Gallo, C., Marioni, G., de Filippis, C. & Lovato, A. Idiopathic Sudden Sensorineural Hearing Loss in Children: A Systematic Review and Meta-analysis. *Otolaryngology–Head and Neck Surgery* **165**, 244–254 (2021).
19. Ho, S., Patel, P., Ballard, D., Rosenfeld, R. & Chandrasekhar, S. Systematic Review and Meta-analysis of Endoscopic vs Microscopic Stapes Surgery for Stapes Fixation. *Otolaryngology–Head and Neck Surgery* **165**, 626–635 (2021).
20. Barbieri, D. *et al.* Autofluorescence and Indocyanine Green in Thyroid Surgery: A Systematic Review and Meta-Analysis. *Laryngoscope* **131**, 1683–1692 (2021).
21. Byun, Y. J., Levy, D. A., Nguyen, S. A., Brennan, E. & Rizk, H. G. Treatment of Vestibular Migraine: A Systematic Review and Meta-analysis. *Laryngoscope* **131**, 186–194 (2021).
22. Wood, J. W., Shaffer, A. D., Kitsko, D. & Chi, D. H. Sudden Sensorineural Hearing Loss in Children—Management and Outcomes: A Meta-analysis. *Laryngoscope* **131**, 425–434 (2021).
23. Coughran, A. *et al.* The Relationship between Croup and Gastroesophageal Reflux: A Systematic Review and Meta-Analysis. *Laryngoscope* **131**, 209–217 (2021).
24. Vinciguerra, A. *et al.* Influence of Surgical Techniques on Endoscopic Dacryocystorhinostomy: A Systematic Review and Meta-analysis. *Otolaryngology–Head and Neck Surgery* **165**, 14–22 (2021).
25. Benito, D. A., Bestourous, D. E., Tong, J. Y., Pasick, L. J. & Sataloff, R. T. Tracheotomy in COVID-19 Patients: A Systematic Review and Meta-analysis of Weaning, Decannulation, and Survival. *Otolaryngology–Head and Neck Surgery* **165**, 398–405 (2021).
26. Simani, L. *et al.* Residual Perforation Risk Assessment of Intratympanic Steroids via Tympanostomy Tube Versus Transtympanic Injections. *Laryngoscope* **131**, E2583–E2591 (2021).

27. Schild, S. D. *et al.* Surgical Management of Sialorrhea: A Systematic Review and Meta-analysis. *Otolaryngology–Head and Neck Surgery* **165**, 507–518 (2021).
28. Timashpolsky, A. *et al.* Management of Type 1 Laryngeal Clefts: A Systematic Review and Meta-analysis. *Otolaryngology–Head and Neck Surgery* **164**, 489–500 (2021).
29. Do, T. M., Unis, G. D., Kattar, N., Ananth, A. & McCoul, E. D. Neuromodulators for Atypical Facial Pain and Neuralgias: A Systematic Review and Meta-Analysis. *Laryngoscope* **131**, 1235–1253 (2021).
30. Mo, S. *et al.* Nasal Peak Inspiratory Flow in Healthy and Obstructed Patients: Systematic Review and Meta-Analysis. *Laryngoscope* **131**, 260–267 (2021).
31. Milinis, K., Thompson, N., Atsmoni, S. C. & Sharma, S. D. Sinogenic Intracranial Suppuration in Children: Systematic Review and Meta-analysis. *Otolaryngology–Head and Neck Surgery* 019459982110438 (2021) doi:10.1177/01945998211043847.
32. Villavisanis, D. F. *et al.* Cochlear Implantation in Meniere's Disease: A Systematic Review and Meta-Analysis. *Laryngoscope* **131**, 1845–1854 (2021).
33. Haddad, R., Ismail, S., Khalaf, M. G. & Matar, N. Lipoinjection for Unilateral Vocal Fold Paralysis Treatment: A Systematic Review and Meta-Analysis. *Laryngoscope* (2021) doi:10.1002/lary.29965.
34. Mair, M. *et al.* A Meta-Analysis of 67 Studies with Presenting Symptoms and Laboratory Tests of COVID-19 Patients. *Laryngoscope* **131**, 1254–1265 (2021).
35. Thorpe, R. K. & Kanotra, S. P. Surgical Management of Bilateral Vocal Fold Paralysis in Children: A Systematic Review and Meta-analysis. *Otolaryngology–Head and Neck Surgery* **164**, 255–263 (2021).
36. Shi, Z. *et al.* Occupational Hearing Loss Associated With Non-Gaussian Noise: A Systematic Review and Meta-analysis. *Ear & Hearing* **42**, 1472–1484 (2021).
37. Nassimizadeh, A., Lancer, H., Hodson, J. & Ahmed, S. Three-Dimensional Endoscopic Endonasal Surgery: A Systematic Review. *Laryngoscope* (2021) doi:10.1002/lary.29939.
38. Medas, F. *et al.* Antibiotic Prophylaxis for Thyroid and Parathyroid Surgery: A Systematic Review and Meta-analysis. *Otolaryngology–Head and Neck Surgery* **164**, 482–488 (2021).
39. Simani, L. *et al.* Paper Patching Versus Watchful Waiting of Traumatic Tympanic Membrane Perforations: A Meta-Analysis. *Laryngoscope* **131**, 2091–2097 (2021).
40. Chen, H. *et al.* Endoscopy-Assisted Transoral Approach to Resect Parapharyngeal Space Tumors: A Systematic Review and Meta-Analysis. *Laryngoscope* **131**, 2246–2253 (2021).
41. Clements, A. C. *et al.* Outcomes of Adenotonsillectomy for Obstructive Sleep Apnea in Prader-Willi Syndrome: Systematic Review and Meta-analysis. *Laryngoscope* **131**, 898–906 (2021).
42. de Virgilio, A. *et al.* Elective neck irradiation in the management of esthesioneuroblastoma: a systematic review and meta-analysis. *Rhinology journal* **0**, 0–0 (2021).
43. Kang, K. *et al.* Effect of Sleep Surgery on C-Reactive Protein Levels in Adults With Obstructive Sleep Apnea: A Meta-Analysis. *Laryngoscope* **131**, 1180–1187 (2021).
44. Kim, D. H., Kim, S. W. & Hwang, S. H. Usefulness of intraoperative frozen section for diagnosing acute invasive fungal rhinosinusitis: A systematic review and meta-analysis. *International Forum of Allergy & Rhinology* **11**, 1347–1354 (2021).

45. Kim, J. Y., Hwang, D., Jang, M., Rhee, C. S. & Han, D. H. Clinical effectiveness of house dust mite immunotherapy in mono- versus poly-sensitised patients with allergic rhinitis: a systematic review and meta-analysis. *Rhinology journal* **0**, 0–0 (2021).
46. Lee, D. S. *et al.* Survival of Young Versus Old Patients With Oral Cavity Squamous Cell Carcinoma: A Meta-Analysis. *Laryngoscope* **131**, 1310–1319 (2021).
47. Reddy, P. D., Yan, F., Nguyen, S. A. & Nathan, C.-A. O. Factors Influencing the Development of Pneumonia in Patients With Head and Neck Cancer: A Meta-analysis. *Otolaryngology–Head and Neck Surgery* **164**, 234–243 (2021).
48. Wong, A., Zhu, D., Kraus, D. & Tham, T. Radiologically Defined Sarcopenia Affects Survival in Head and Neck Cancer: A Meta-Analysis. *Laryngoscope* **131**, 333–341 (2021).
49. Kao, Y., Huang, Y., Tsai, S., Lai, M. & Kang, Y. Effects of implants with steroids after endoscopic sinus surgery: A systematic review with meta-analysis of randomized controlled trials. *International Forum of Allergy & Rhinology* **11**, 1663–1675 (2021).
50. Tsabouri, S. *et al.* Omalizumab for the treatment of allergic rhinitis: a systematic review and meta-analysis. *Rhinology journal* **0**, 0–0 (2021).
51. Yin, M. *et al.* Effect of Endoscope Sinus Surgery on Pulmonary Function in Cystic Fibrosis Patients: A Meta-Analysis. *Laryngoscope* **131**, 720–725 (2021).
52. Chaaban, M. R. *et al.* Meta-analysis Exploring Sinopulmonary Outcomes of Aspirin Desensitization in Aspirin-Exacerbated Respiratory Disease. *Otolaryngology–Head and Neck Surgery* **164**, 11–18 (2021).
53. Ab Shukor, N. F., Han, W., Lee, J. & Seo, Y. J. Crucial Music Components Needed for Speech Perception Enhancement of Pediatric Cochlear Implant Users: A Systematic Review and Meta-Analysis. *Audiology and Neurotology* **26**, 389–413 (2021).
54. Bollig, C. A. *et al.* Systematic Review of Second Primary Oropharyngeal Cancers in Patients With p16+ Oropharyngeal Cancer. *Otolaryngology–Head and Neck Surgery* **164**, 733–740 (2021).
55. Soh, T. C. F., Ong, Q. J. & Yip, H. M. Complications of Neck Drains in Thyroidectomies: A Systematic Review and Meta-Analysis. *Laryngoscope* **131**, 690–700 (2021).
56. Strum, D. *et al.* Prevalence of Sensorineural Hearing Loss in Pediatric Patients with Sickle Cell Disease: A Meta-analysis. *Laryngoscope* **131**, 1147–1156 (2021).
57. Benchetrit, L., Torabi, S. J., Givi, B., Haughey, B. & Judson, B. L. Prognostic Significance of Extranodal Extension in HPV-Mediated Oropharyngeal Carcinoma: A Systematic Review and Meta-analysis. *Otolaryngology–Head and Neck Surgery* **164**, 720–732 (2021).
58. Stepp, W. H. *et al.* HPV in the malignant transformation of sinonasal inverted papillomas: A meta-analysis. *International Forum of Allergy & Rhinology* **11**, 1461–1471 (2021).
59. Cabrera, C. I., Joseph Jones, A., Philleo Parker, N., Emily Lynn Blevins, A. & Weidenbecher, M. S. Pectoralis Major Onlay vs Interpositional Reconstruction Fistulation After Salvage Total Laryngectomy: Systematic Review and Meta-analysis. *Otolaryngology–Head and Neck Surgery* **164**, 972–983 (2021).
60. Goel, A. N., Omorogbe, A., Hackett, A., Rothschild, M. A. & Londino, A. v. Risk Factors for Multiple Tympanostomy Tube Placements in Children: Systematic Review and Meta-Analysis. *Laryngoscope* **131**, E2363–E2370 (2021).
61. Jalali, M. M., Soleimani, R., Soltanipour, S. & Jalali, S. M. Pharmacological Treatments of Bell's Palsy in Adults: A Systematic Review and Network Meta-Analysis. *Laryngoscope* **131**, 1615–1625 (2021).
62. Rodrigues, J. *et al.* Anxiety and depression risk in patients with allergic rhinitis: a systematic review and meta-analysis. *Rhinology journal* **0**, 0–0 (2021).

63. Wu, Y. *et al.* Slide Tracheoplasty for Congenital Tracheal Stenosis Repair: A Systematic Review and Meta-Analysis. *Laryngoscope* (2021) doi:10.1002/lary.29771.
64. Ding, R., Sun, Q. & Wang, Y. Association Between Human Papilloma Virus Infection and Malignant Sinonasal Inverted Papilloma. *Laryngoscope* **131**, 1200–1205 (2021).
65. Walia, A. *et al.* Management of Flap Failure After Head and Neck Reconstruction: A Systematic Review and Meta-analysis. *Otolaryngology–Head and Neck Surgery* 019459982110446 (2021) doi:10.1177/01945998211044683.
66. Bauer, E. *et al.* Complications After Soft Tissue With Plate vs Bony Mandibular Reconstruction: A Systematic Review and Meta-analysis. *Otolaryngology–Head and Neck Surgery* **164**, 501–511 (2021).
67. Obrero-Gaitán, E. *et al.* Misperception of Visual Vertical in Peripheral Vestibular Disorders. A Systematic Review With Meta-Analysis. *Laryngoscope* **131**, 1110–1121 (2021).
68. Ju, X. *et al.* High-Risk Human Papillomavirus–Related Oropharyngeal Squamous Cell Carcinoma Among Non-Indigenous and Indigenous Populations: A Systematic Review. *Otolaryngology–Head and Neck Surgery* **165**, 23–32 (2021).
69. Titirungruang, C. K., Charakorn, N., Chaitusaney, B. & Hirunwiwatkul, P. Is postoperative nasal packing after septoplasty safe? A systematic review and meta-analysis of randomized controlled studies. *Rhinology journal* **59**, 340–351 (2021).
70. Allen, M. *et al.* Tracheal Resection in the Management of Thyroid Cancer: An Evidence-Based Approach. *Laryngoscope* **131**, 932–946 (2021).
71. de Virgilio, A. *et al.* Different Surgical Strategies in the Prevention of Frey Syndrome: A Systematic Review and Meta-analysis. *Laryngoscope* **131**, 1761–1768 (2021).
72. Saniasiaya, J., Islam, M. A. & Abdullah, B. Prevalence and Characteristics of Taste Disorders in Cases of COVID-19: A Meta-analysis of 29,349 Patients. *Otolaryngology–Head and Neck Surgery* **165**, 33–42 (2021).
73. Williams, Z. J., Suzman, E. & Woynaroski, T. G. Prevalence of Decreased Sound Tolerance (Hyperacusis) in Individuals With Autism Spectrum Disorder. *Ear & Hearing* 1137–1150 (2021) doi:10.1097/AUD.0000000000001005.
74. Saniasiaya, J., Islam, M. A. & Abdullah, B. Prevalence of Olfactory Dysfunction in Coronavirus Disease 2019 (COVID-19): A Meta-analysis of 27,492 Patients. *Laryngoscope* **131**, 865–878 (2021).
75. Kim, D. H., Kim, Y., Kim, S. W. & Hwang, S. H. Usefulness of Sentinel Lymph Node Biopsy for Oral Cancer: A Systematic Review and Meta-Analysis. *Laryngoscope* **131**, E459–E465 (2021).
